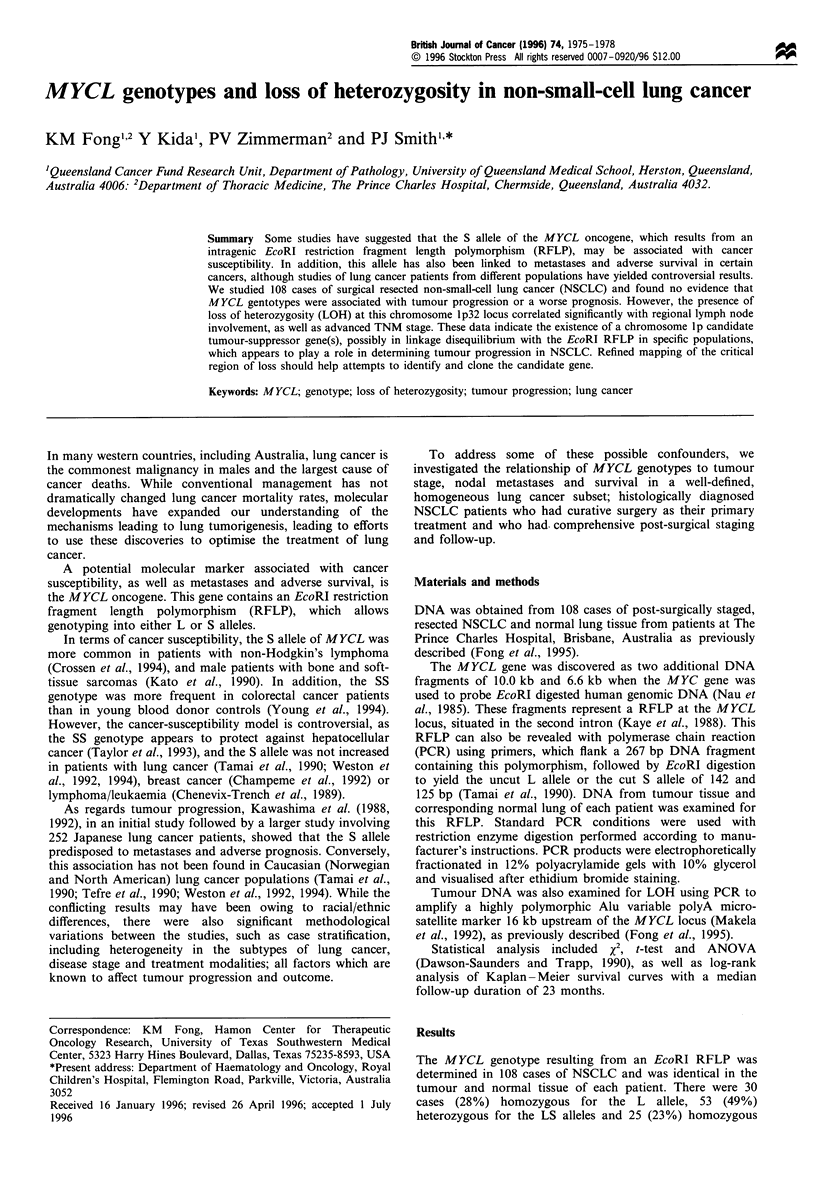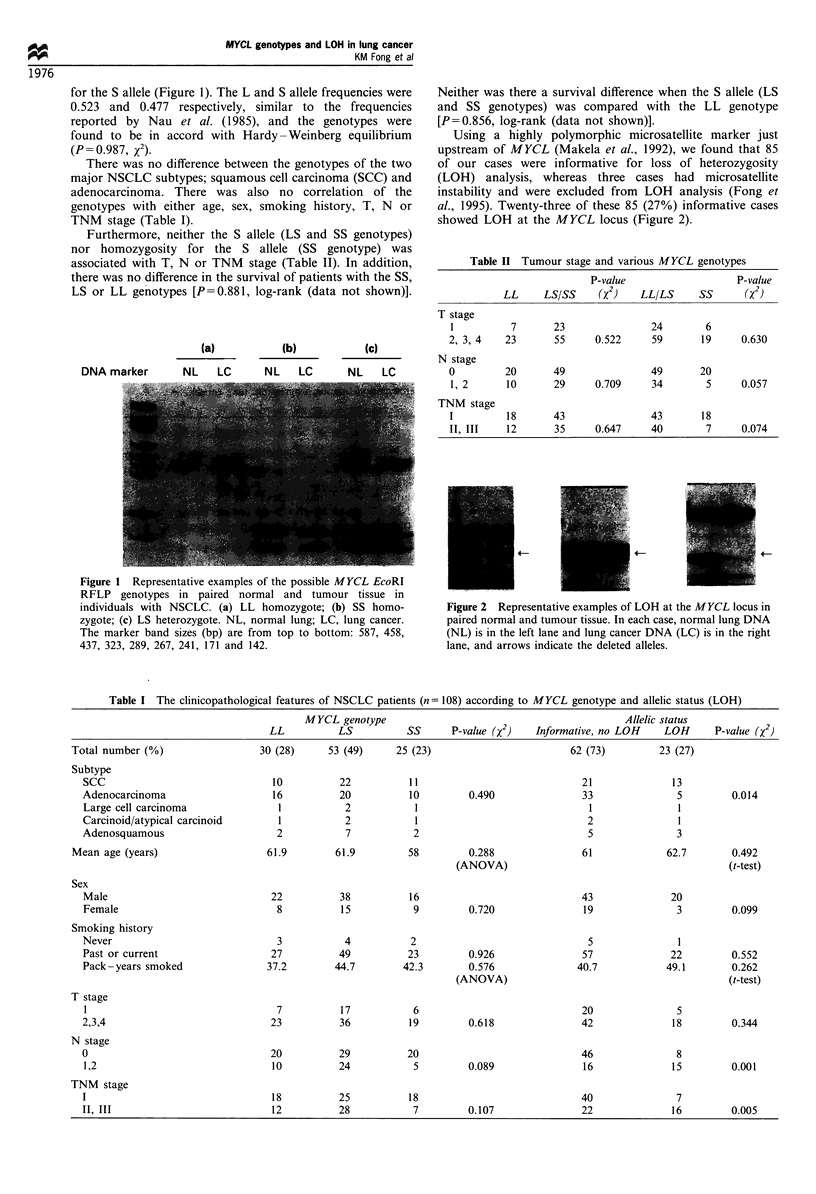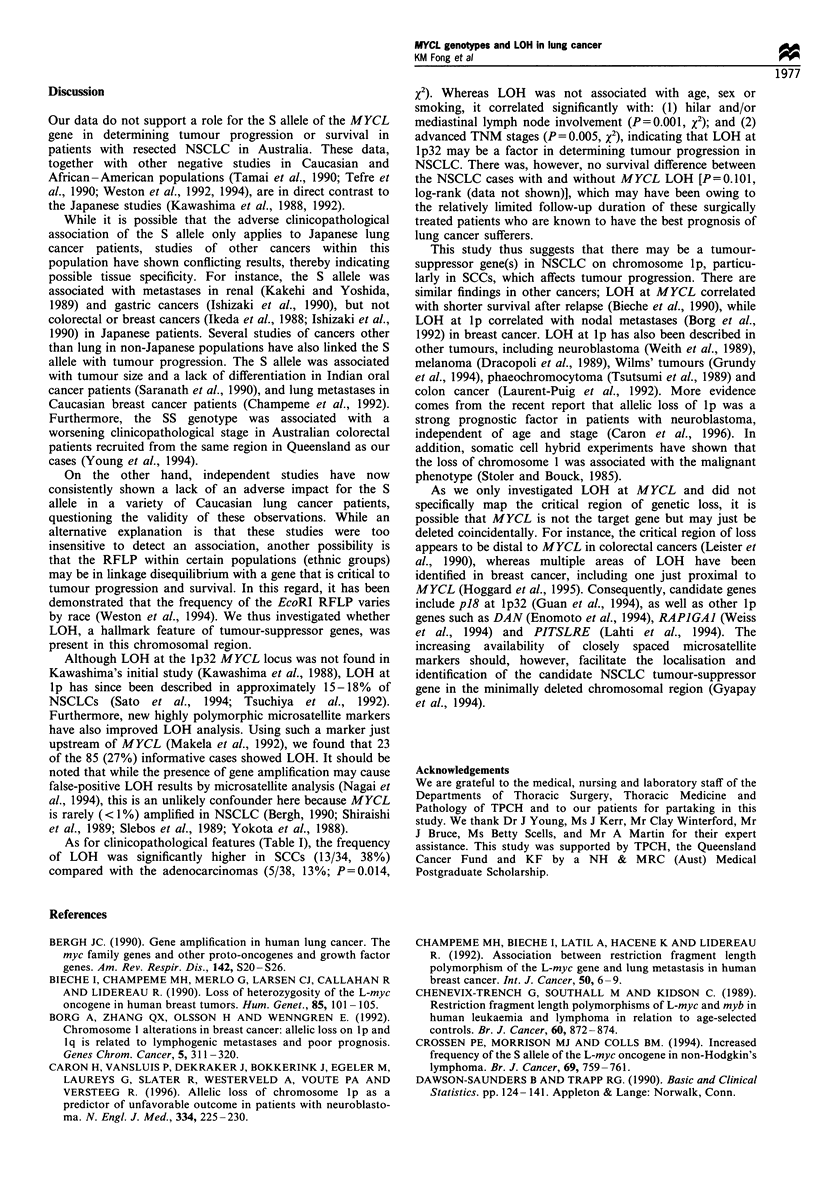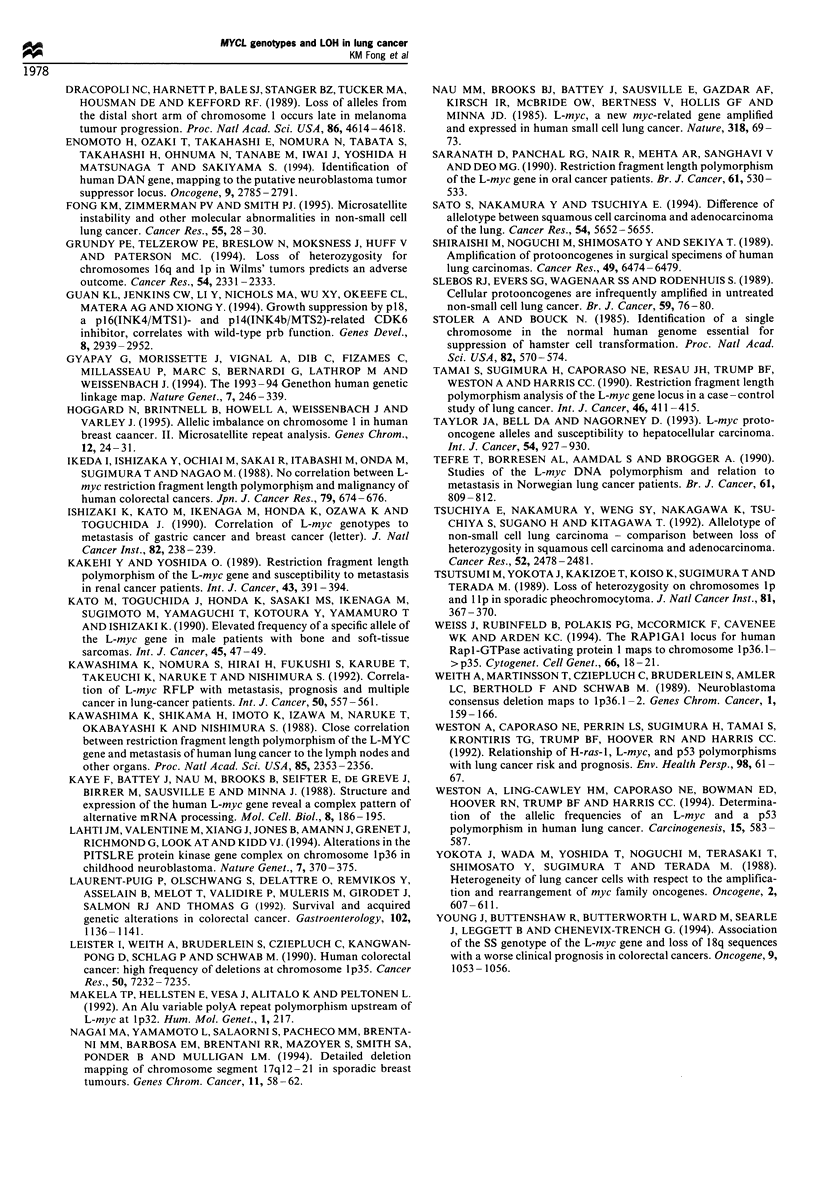# MYCL genotypes and loss of heterozygosity in non-small-cell lung cancer.

**DOI:** 10.1038/bjc.1996.662

**Published:** 1996-12

**Authors:** K. M. Fong, Y. Kida, P. V. Zimmerman, P. J. Smith

**Affiliations:** Department of Pathology, University of Queensland Medical School, Herston, Australia.

## Abstract

**Images:**


British Journal of Cancer (1996) 74, 1975-1978
© 1996 Stockton Press All rights reserved 0007-0920/96 $12.00

### *MYCL* genotypes and loss of heterozygosity in non-small-cell lung cancer

KM Fong[1,2] Y Kida[1], PV Zimmerman[2] and PJ Smith[1,*]

[1]*Queensland Cancer Fund Research Unit, Department of Pathology, University of Queensland Medical School, Herston, Queensland, Australia 4006:* [2]*Department of Thoracic Medicine, The Prince Charles Hospital, Chermside, Queensland, Australia 4032.*

**Summary** Some studies have suggested that the S allele of the *MYCL* oncogene, which results from an intragenic *Eco*RI restriction fragment length polymorphism (RFLP), may be associated with cancer susceptibility. In addition, this allele has also been linked to metastases and adverse survival in certain cancers, although studies of lung cancer patients from different populations have yielded controversial results. We studied 108 cases of surgical resected non-small-cell lung cancer (NSCLC) and found no evidence that *MYCL* gentotypes were associated with tumour progression or a worse prognosis. However, the presence of loss of heterozygosity (LOH) at this chromosome 1p32 locus correlated significantly with regional lymph node involvement, as well as advanced TNM stage. These data indicate the existence of a chromosome 1p candidate tumour-suppressor gene(s), possibly in linkage disequilibrium with the *Eco*RI RFLP in specific populations, which appears to play a role in determining tumour progression in NSCLC. Refined mapping of the critical region of loss should help attempts to identify and clone the candidate gene.

**Keywords:** *MYCL*; genotype; loss of heterozygosity; tumour progression; lung cancer

In many western countries, including Australia, lung cancer is the commonest malignancy in males and the largest cause of cancer deaths. While conventional management has not dramatically changed lung cancer mortality rates, molecular developments have expanded our understanding of the mechanisms leading to lung tumorigenesis, leading to efforts to use these discoveries to optimise the treatment of lung cancer.

A potential molecular marker associated with cancer susceptibility, as well as metastases and adverse survival, is the *MYCL* oncogene. This gene contains an *Eco*RI restriction fragment length polymorphism (RFLP), which allows genotyping into either L or S alleles.

In terms of cancer susceptibility, the S allele of *MYCL* was more common in patients with non-Hodgkin's lymphoma (Crossen *et al.*, 1994), and male patients with bone and soft-tissue sarcomas (Kato *et al.*, 1990). In addition, the SS genotype was more frequent in colorectal cancer patients than in young blood donor controls (Young *et al.*, 1994). However, the cancer-susceptibility model is controversial, as the SS genotype appears to protect against hepatocellular cancer (Taylor *et al.*, 1993), and the S allele was not increased in patients with lung cancer (Tamai *et al.*, 1990; Weston *et al.*, 1992, 1994), breast cancer (Champeme *et al.*, 1992) or lymphoma/leukaemia (Chenevix-Trench *et al.*, 1989).

As regards tumour progression, Kawashima *et al.* (1988, 1992), in an initial study followed by a larger study involving 252 Japanese lung cancer patients, showed that the S allele predisposed to metastases and adverse prognosis. Conversely, this association has not been found in Caucasian (Norwegian and North American) lung cancer populations (Tamai *et al.*, 1990; Tefre *et al.*, 1990; Weston *et al.*, 1992, 1994). While the conflicting results may have been owing to racial/ethnic differences, there were also significant methodological variations between the studies, such as case stratification, including heterogeneity in the subtypes of lung cancer, disease stage and treatment modalities; all factors which are known to affect tumour progression and outcome.

Correspondence: KM Fong, Hamon Center for Therapeutic Oncology Research, University of Texas Southwestern Medical Center, 5323 Harry Hines Boulevard, Dallas, Texas 75235-8593, USA
*Present address: Department of Haematology and Oncology, Royal Children's Hospital, Flemington Road, Parkville, Victoria, Australia 3052
Received 16 January 1996; revised 26 April 1996; accepted 1 July 1996

To address some of these possible confounders, we investigated the relationship of *MYCL* genotypes to tumour stage, nodal metastases and survival in a well-defined, homogeneous lung cancer subset; histologically diagnosed NSCLC patients who had curative surgery as their primary treatment and who had comprehensive post-surgical staging and follow-up.

### Materials and methods

DNA was obtained from 108 cases of post-surgically staged, resected NSCLC and normal lung tissue from patients at The Prince Charles Hospital, Brisbane, Australia as previously described (Fong *et al.*, 1995).

The *MYCL* gene was discovered as two additional DNA fragments of 10.0 kb and 6.6 kb when the *MYC* gene was used to probe *Eco*RI digested human genomic DNA (Nau *et al.*, 1985). These fragments represent a RFLP at the *MYCL* locus, situated in the second intron (Kaye *et al.*, 1988). This RFLP can also be revealed with polymerase chain reaction (PCR) using primers, which flank a 267 bp DNA fragment containing this polymorphism, followed by *Eco*RI digestion to yield the uncut L allele or the cut S allele of 142 and 125 bp (Tamai *et al.*, 1990). DNA from tumour tissue and corresponding normal lung of each patient was examined for this RFLP. Standard PCR conditions were used with restriction enzyme digestion performed according to manufacturer's instructions. PCR products were electrophoretically fractionated in 12% polyacrylamide gels with 10% glycerol and visualised after ethidium bromide staining.

Tumour DNA was also examined for LOH using PCR to amplify a highly polymorphic Alu variable polyA microsatellite marker 16 kb upstream of the *MYCL* locus (Makela *et al.*, 1992), as previously described (Fong *et al.*, 1995).

Statistical analysis included $\chi^2$, *t*-test and ANOVA (Dawson-Saunders and Trapp, 1990), as well as log-rank analysis of Kaplan–Meier survival curves with a median follow-up duration of 23 months.

### Results

The *MYCL* genotype resulting from an *Eco*RI RFLP was determined in 108 cases of NSCLC and was identical in the tumour and normal tissue of each patient. There were 30 cases (28%) homozygous for the L allele, 53 (49%) heterozygous for the LS alleles and 25 (23%) homozygous for the S allele (Figure 1). The L and S allele frequencies were 0.523 and 0.477 respectively, similar to the frequencies reported by Nau *et al.* (1985), and the genotypes were found to be in accord with Hardy–Weinberg equilibrium ($P=0.987$, $\chi^2$).

There was no difference between the genotypes of the two major NSCLC subtypes; squamous cell carcinoma (SCC) and adenocarcinoma. There was also no correlation of the genotypes with either age, sex, smoking history, T, N or TNM stage (Table I).

Furthermore, neither the S allele (LS and SS genotypes) nor homozygosity for the S allele (SS genotype) was associated with T, N or TNM stage (Table II). In addition, there was no difference in the survival of patients with the SS, LS or LL genotypes [$P=0.881$, log-rank (data not shown)]. Neither was there a survival difference when the S allele (LS and SS genotypes) was compared with the LL genotype [$P=0.856$, log-rank (data not shown)].

Using a highly polymorphic microsatellite marker just upstream of *MYCL* (Makela *et al.*, 1992), we found that 85 of our cases were informative for loss of heterozygosity (LOH) analysis, whereas three cases had microsatellite instability and were excluded from LOH analysis (Fong *et al.*, 1995). Twenty-three of these 85 (27%) informative cases showed LOH at the *MYCL* locus (Figure 2).

**Figure 1** Representative examples of the possible *MYCL EcoRI* RFLP genotypes in paired normal and tumour tissue in individuals with NSCLC. (**a**) LL homozygote; (**b**) SS homozygote; (**c**) LS heterozygote. NL, normal lung; LC, lung cancer. The marker band sizes (bp) are from top to bottom: 587, 458, 437, 323, 289, 267, 241, 171 and 142.

**Table II** Tumour stage and various *MYCL* genotypes

| | *LL* | *LS/SS* | P-*value* ($\chi^2$) | *LL/LS* | *SS* | P-*value* ($\chi^2$) |
|---|---|---|---|---|---|---|
| T stage | | | | | | |
| 1 | 7 | 23 | | 24 | 6 | |
| 2, 3, 4 | 23 | 55 | 0.522 | 59 | 19 | 0.630 |
| N stage | | | | | | |
| 0 | 20 | 49 | | 49 | 20 | |
| 1, 2 | 10 | 29 | 0.709 | 34 | 5 | 0.057 |
| TNM stage | | | | | | |
| I | 18 | 43 | | 43 | 18 | |
| II, III | 12 | 35 | 0.647 | 40 | 7 | 0.074 |

**Figure 2** Representative examples of LOH at the *MYCL* locus in paired normal and tumour tissue. In each case, normal lung DNA (NL) is in the left lane and lung cancer DNA (LC) is in the right lane, and arrows indicate the deleted alleles.

**Table I** The clinicopathological features of NSCLC patients ($n=108$) according to *MYCL* genotype and allelic status (LOH)

| | *MYCL genotype* *LL* | *LS* | *SS* | P-*value* ($\chi^2$) | *Allelic status* *Informative, no LOH* | *LOH* | P-*value* ($\chi^2$) |
|---|---|---|---|---|---|---|---|
| Total number (%) | 30 (28) | 53 (49) | 25 (23) | | 62 (73) | 23 (27) | |
| Subtype | | | | | | | |
| SCC | 10 | 22 | 11 | | 21 | 13 | |
| Adenocarcinoma | 16 | 20 | 10 | 0.490 | 33 | 5 | 0.014 |
| Large cell carcinoma | 1 | 2 | 1 | | 1 | 1 | |
| Carcinoid/atypical carcinoid | 1 | 2 | 1 | | 2 | 1 | |
| Adenosquamous | 2 | 7 | 2 | | 5 | 3 | |
| Mean age (years) | 61.9 | 61.9 | 58 | 0.288 (ANOVA) | 61 | 62.7 | 0.492 (*t*-test) |
| Sex | | | | | | | |
| Male | 22 | 38 | 16 | | 43 | 20 | |
| Female | 8 | 15 | 9 | 0.720 | 19 | 3 | 0.099 |
| Smoking history | | | | | | | |
| Never | 3 | 4 | 2 | | 5 | 1 | |
| Past or current | 27 | 49 | 23 | 0.926 | 57 | 22 | 0.552 |
| Pack–years smoked | 37.2 | 44.7 | 42.3 | 0.576 (ANOVA) | 40.7 | 49.1 | 0.262 (*t*-test) |
| T stage | | | | | | | |
| 1 | 7 | 17 | 6 | | 20 | 5 | |
| 2,3,4 | 23 | 36 | 19 | 0.618 | 42 | 18 | 0.344 |
| N stage | | | | | | | |
| 0 | 20 | 29 | 20 | | 46 | 8 | |
| 1,2 | 10 | 24 | 5 | 0.089 | 16 | 15 | 0.001 |
| TNM stage | | | | | | | |
| I | 18 | 25 | 18 | | 40 | 7 | |
| II, III | 12 | 28 | 7 | 0.107 | 22 | 16 | 0.005 |

### Discussion

Our data do not support a role for the S allele of the *MYCL* gene in determining tumour progression or survival in patients with resected NSCLC in Australia. These data, together with other negative studies in Caucasian and African-American populations (Tamai *et al.*, 1990; Tefre *et al.*, 1990; Weston *et al.*, 1992, 1994), are in direct contrast to the Japanese studies (Kawashima *et al.*, 1988, 1992).

While it is possible that the adverse clinicopathological association of the S allele only applies to Japanese lung cancer patients, studies of other cancers within this population have shown conflicting results, thereby indicating possible tissue specificity. For instance, the S allele was associated with metastases in renal (Kakehi and Yoshida, 1989) and gastric cancers (Ishizaki *et al.*, 1990), but not colorectal or breast cancers (Ikeda *et al.*, 1988; Ishizaki *et al.*, 1990) in Japanese patients. Several studies of cancers other than lung in non-Japanese populations have also linked the S allele with tumour progression. The S allele was associated with tumour size and a lack of differentiation in Indian oral cancer patients (Saranath *et al.*, 1990), and lung metastases in Caucasian breast cancer patients (Champeme *et al.*, 1992). Furthermore, the SS genotype was associated with a worsening clinicopathological stage in Australian colorectal patients recruited from the same region in Queensland as our cases (Young *et al.*, 1994).

On the other hand, independent studies have now consistently shown a lack of an adverse impact for the S allele in a variety of Caucasian lung cancer patients, questioning the validity of these observations. While an alternative explanation is that these studies were too insensitive to detect an association, another possibility is that the RFLP within certain populations (ethnic groups) may be in linkage disequilibrium with a gene that is critical to tumour progression and survival. In this regard, it has been demonstrated that the frequency of the *Eco*RI RFLP varies by race (Weston *et al.*, 1994). We thus investigated whether LOH, a hallmark feature of tumour-suppressor genes, was present in this chromosomal region.

Although LOH at the 1p32 *MYCL* locus was not found in Kawashima's initial study (Kawashima *et al.*, 1988), LOH at 1p has since been described in approximately 15-18% of NSCLCs (Sato *et al.*, 1994; Tsuchiya *et al.*, 1992). Furthermore, new highly polymorphic microsatellite markers have also improved LOH analysis. Using such a marker just upstream of *MYCL* (Makela *et al.*, 1992), we found that 23 of the 85 (27%) informative cases showed LOH. It should be noted that while the presence of gene amplification may cause false-positive LOH results by microsatellite analysis (Nagai *et al.*, 1994), this is an unlikely confounder here because *MYCL* is rarely (<1%) amplified in NSCLC (Bergh, 1990; Shiraishi *et al.*, 1989; Slebos *et al.*, 1989; Yokota *et al.*, 1988).

As for clinicopathological features (Table I), the frequency of LOH was significantly higher in SCCs (13/34, 38%) compared with the adenocarcinomas (5/38, 13%; $P=0.014$, $\chi^2$). Whereas LOH was not associated with age, sex or smoking, it correlated significantly with: (1) hilar and/or mediastinal lymph node involvement ($P=0.001$, $\chi^2$); and (2) advanced TNM stages ($P=0.005$, $\chi^2$), indicating that LOH at 1p32 may be a factor in determining tumour progression in NSCLC. There was, however, no survival difference between the NSCLC cases with and without *MYCL* LOH [$P=0.101$, log-rank (data not shown)], which may have been owing to the relatively limited follow-up duration of these surgically treated patients who are known to have the best prognosis of lung cancer sufferers.

This study thus suggests that there may be a tumour-suppressor gene(s) in NSCLC on chromosome 1p, particularly in SCCs, which affects tumour progression. There are similar findings in other cancers; LOH at *MYCL* correlated with shorter survival after relapse (Bieche *et al.*, 1990), while LOH at 1p correlated with nodal metastases (Borg *et al.*, 1992) in breast cancer. LOH at 1p has also been described in other tumours, including neuroblastoma (Weith *et al.*, 1989), melanoma (Dracopoli *et al.*, 1989), Wilms' tumours (Grundy *et al.*, 1994), phaeochromocytoma (Tsutsumi *et al.*, 1989) and colon cancer (Laurent-Puig *et al.*, 1992). More evidence comes from the recent report that allelic loss of 1p was a strong prognostic factor in patients with neuroblastoma, independent of age and stage (Caron *et al.*, 1996). In addition, somatic cell hybrid experiments have shown that the loss of chromosome 1 was associated with the malignant phenotype (Stoler and Bouck, 1985).

As we only investigated LOH at *MYCL* and did not specifically map the critical region of genetic loss, it is possible that *MYCL* is not the target gene but may just be deleted coincidentally. For instance, the critical region of loss appears to be distal to *MYCL* in colorectal cancers (Leister *et al.*, 1990), whereas multiple areas of LOH have been identified in breast cancer, including one just proximal to *MYCL* (Hoggard *et al.*, 1995). Consequently, candidate genes include *p18* at 1p32 (Guan *et al.*, 1994), as well as other 1p genes such as *DAN* (Enomoto *et al.*, 1994), *RAP1GA1* (Weiss *et al.*, 1994) and *PITSLRE* (Lahti *et al.*, 1994). The increasing availability of closely spaced microsatellite markers should, however, facilitate the localisation and identification of the candidate NSCLC tumour-suppressor gene in the minimally deleted chromosomal region (Gyapay *et al.*, 1994).

**Acknowledgements**
We are grateful to the medical, nursing and laboratory staff of the Departments of Thoracic Surgery, Thoracic Medicine and Pathology of TPCH and to our patients for partaking in this study. We thank Dr J Young, Ms J Kerr, Mr Clay Winterford, Mr J Bruce, Ms Betty Scells, and Mr A Martin for their expert assistance. This study was supported by TPCH, the Queensland Cancer Fund and KF by a NH & MRC (Aust) Medical Postgraduate Scholarship.

### References

BERGH JC. (1990). Gene amplification in human lung cancer. The *myc* family genes and other proto-oncogenes and growth factor genes. *Am. Rev. Respir. Dis.*, **142**, S20-S26.

BIECHE I, CHAMPEME MH, MERLO G, LARSEN CJ, CALLAHAN R AND LIDEREAU R. (1990). Loss of heterozygosity of the L-*myc* oncogene in human breast tumors. *Hum. Genet.*, **85**, 101-105.

BORG A, ZHANG QX, OLSSON H AND WENNGREN E. (1992). Chromosome 1 alterations in breast cancer: allelic loss on 1p and 1q is related to lymphogenic metastases and poor prognosis. *Genes Chrom. Cancer*, **5**, 311-320.

CARON H, VANSLUIS P, DEKRAKER J, BOKKERINK J, EGELER M, LAUREYS G, SLATER R, WESTERVELD A, VOUTE PA AND VERSTEEG R. (1996). Allelic loss of chromosome 1p as a predictor of unfavorable outcome in patients with neuroblastoma. *N. Engl. J. Med.*, **334**, 225-230.

CHAMPEME MH, BIECHE I, LATIL A, HACENE K AND LIDEREAU R. (1992). Association between restriction fragment length polymorphism of the L-*myc* gene and lung metastasis in human breast cancer. *Int. J. Cancer*, **50**, 6-9.

CHENEVIX-TRENCH G, SOUTHALL M AND KIDSON C. (1989). Restriction fragment length polymorphisms of L-*myc* and *myb* in human leukaemia and lymphoma in relation to age-selected controls. *Br. J. Cancer*, **60**, 872-874.

CROSSEN PE, MORRISON MJ AND COLLS BM. (1994). Increased frequency of the S allele of the L-*myc* oncogene in non-Hodgkin's lymphoma. *Br. J. Cancer*, **69**, 759-761.

DAWSON-SAUNDERS B AND TRAPP RG. (1990). *Basic and Clinical Statistics*. pp. 124-141. Appleton & Lange: Norwalk, Conn.

DRACOPOLI NC, HARNETT P, BALE SJ, STANGER BZ, TUCKER MA, HOUSMAN DE AND KEFFORD RF. (1989). Loss of alleles from the distal short arm of chromosome 1 occurs late in melanoma tumour progression. *Proc. Natl Acad. Sci. USA*, **86**, 4614–4618.

ENOMOTO H, OZAKI T, TAKAHASHI E, NOMURA N, TABATA S, TAKAHASHI H, OHNUMA N, TANABE M, IWAI J, YOSHIDA H MATSUNAGA T AND SAKIYAMA S. (1994). Identification of human DAN gene, mapping to the putative neuroblastoma tumor suppressor locus. *Oncogene*, **9**, 2785–2791.

FONG KM, ZIMMERMAN PV AND SMITH PJ. (1995). Microsatellite instability and other molecular abnormalities in non-small cell lung cancer. *Cancer Res.*, **55**, 28–30.

GRUNDY PE, TELZEROW PE, BRESLOW N, MOKSNESS J, HUFF V AND PATERSON MC. (1994). Loss of heterozygosity for chromosomes 16q and 1p in Wilms' tumors predicts an adverse outcome. *Cancer Res.*, **54**, 2331–2333.

GUAN KL, JENKINS CW, LI Y, NICHOLS MA, WU XY, OKEEFE CL, MATERA AG AND XIONG Y. (1994). Growth suppression by p18, a p16(INK4/MTS1)- and p14(INK4b/MTS2)-related CDK6 inhibitor, correlates with wild-type prb function. *Genes Devel.*, **8**, 2939–2952.

GYAPAY G, MORISSETTE J, VIGNAL A, DIB C, FIZAMES C, MILLASSEAU P, MARC S, BERNARDI G, LATHROP M AND WEISSENBACH J. (1994). The 1993–94 Genethon human genetic linkage map. *Nature Genet.*, **7**, 246–339.

HOGGARD N, BRINTNELL B, HOWELL A, WEISSENBACH J AND VARLEY J. (1995). Allelic imbalance on chromosome 1 in human breast caancer. II. Microsatellite repeat analysis. *Genes Chrom.*, **12**, 24–31.

IKEDA I, ISHIZAKA Y, OCHIAI M, SAKAI R, ITABASHI M, ONDA M, SUGIMURA T AND NAGAO M. (1988). No correlation between L-*myc* restriction fragment length polymorphism and malignancy of human colorectal cancers. *Jpn. J. Cancer Res.*, **79**, 674–676.

ISHIZAKI K, KATO M, IKENAGA M, HONDA K, OZAWA K AND TOGUCHIDA J. (1990). Correlation of L-*myc* genotypes to metastasis of gastric cancer and breast cancer (letter). *J. Natl Cancer Inst.*, **82**, 238–239.

KAKEHI Y AND YOSHIDA O. (1989). Restriction fragment length polymorphism of the L-*myc* gene and susceptibility to metastasis in renal cancer patients. *Int. J. Cancer*, **43**, 391–394.

KATO M, TOGUCHIDA J, HONDA K, SASAKI MS, IKENAGA M, SUGIMOTO M, YAMAGUCHI T, KOTOURA Y, YAMAMURO T AND ISHIZAKI K. (1990). Elevated frequency of a specific allele of the L-*myc* gene in male patients with bone and soft-tissue sarcomas. *Int. J. Cancer*, **45**, 47–49.

KAWASHIMA K, NOMURA S, HIRAI H, FUKUSHI S, KARUBE T, TAKEUCHI K, NARUKE T AND NISHIMURA S. (1992). Correlation of L-*myc* RFLP with metastasis, prognosis and multiple cancer in lung-cancer patients. *Int. J. Cancer*, **50**, 557–561.

KAWASHIMA K, SHIKAMA H, IMOTO K, IZAWA M, NARUKE T, OKABAYASHI K AND NISHIMURA S. (1988). Close correlation between restriction fragment length polymorphism of the L-MYC gene and metastasis of human lung cancer to the lymph nodes and other organs. *Proc. Natl Acad. Sci. USA*, **85**, 2353–2356.

KAYE F, BATTEY J, NAU M, BROOKS B, SEIFTER E, DE GREVE J, BIRRER M, SAUSVILLE E AND MINNA J. (1988). Structure and expression of the human L-*myc* gene reveal a complex pattern of alternative mRNA processing. *Mol. Cell. Biol.*, **8**, 186–195.

LAHTI JM, VALENTINE M, XIANG J, JONES B, AMANN J, GRENET J, RICHMOND G, LOOK AT AND KIDD VJ. (1994). Alterations in the PITSLRE protein kinase gene complex on chromosome 1p36 in childhood neuroblastoma. *Nature Genet.*, **7**, 370–375.

LAURENT-PUIG P, OLSCHWANG S, DELATTRE O, REMVIKOS Y, ASSELAIN B, MELOT T, VALIDIRE P, MULERIS M, GIRODET J, SALMON RJ AND THOMAS G (1992). Survival and acquired genetic alterations in colorectal cancer. *Gastroenterology*, **102**, 1136–1141.

LEISTER I, WEITH A, BRUDERLEIN S, CZIEPLUCH C, KANGWANPONG D, SCHLAG P AND SCHWAB M. (1990). Human colorectal cancer: high frequency of deletions at chromosome 1p35. *Cancer Res.*, **50**, 7232–7235.

MAKELA TP, HELLSTEN E, VESA J, ALITALO K AND PELTONEN L. (1992). An Alu variable polyA repeat polymorphism upstream of L-*myc* at 1p32. *Hum. Mol. Genet.*, **1**, 217.

NAGAI MA, YAMAMOTO L, SALAORNI S, PACHECO MM, BRENTANI MM, BARBOSA EM, BRENTANI RR, MAZOYER S, SMITH SA, PONDER B AND MULLIGAN LM. (1994). Detailed deletion mapping of chromosome segment 17q12–21 in sporadic breast tumours. *Genes Chrom. Cancer*, **11**, 58–62.

NAU MM, BROOKS BJ, BATTEY J, SAUSVILLE E, GAZDAR AF, KIRSCH IR, MCBRIDE OW, BERTNESS V, HOLLIS GF AND MINNA JD. (1985). L-*myc*, a new *myc*-related gene amplified and expressed in human small cell lung cancer. *Nature*, **318**, 69–73.

SARANATH D, PANCHAL RG, NAIR R, MEHTA AR, SANGHAVI V AND DEO MG. (1990). Restriction fragment length polymorphism of the L-*myc* gene in oral cancer patients. *Br. J. Cancer*, **61**, 530–533.

SATO S, NAKAMURA Y AND TSUCHIYA E. (1994). Difference of allelotype between squamous cell carcinoma and adenocarcinoma of the lung. *Cancer Res.*, **54**, 5652–5655.

SHIRAISHI M, NOGUCHI M, SHIMOSATO Y AND SEKIYA T. (1989). Amplification of protooncogenes in surgical specimens of human lung carcinomas. *Cancer Res.*, **49**, 6474–6479.

SLEBOS RJ, EVERS SG, WAGENAAR SS AND RODENHUIS S. (1989). Cellular protooncogenes are infrequently amplified in untreated non-small cell lung cancer. *Br. J. Cancer*, **59**, 76–80.

STOLER A AND BOUCK N. (1985). Identification of a single chromosome in the normal human genome essential for suppression of hamster cell transformation. *Proc. Natl Acad. Sci. USA*, **82**, 570–574.

TAMAI S, SUGIMURA H, CAPORASO NE, RESAU JH, TRUMP BF, WESTON A AND HARRIS CC. (1990). Restriction fragment length polymorphism analysis of the L-*myc* gene locus in a case–control study of lung cancer. *Int. J. Cancer*, **46**, 411–415.

TAYLOR JA, BELL DA AND NAGORNEY D. (1993). L-*myc* proto-oncogene alleles and susceptibility to hepatocellular carcinoma. *Int. J. Cancer*, **54**, 927–930.

TEFRE T, BORRESEN AL, AAMDAL S AND BROGGER A. (1990). Studies of the L-*myc* DNA polymorphism and relation to metastasis in Norwegian lung cancer patients. *Br. J. Cancer*, **61**, 809–812.

TSUCHIYA E, NAKAMURA Y, WENG SY, NAKAGAWA K, TSUCHIYA S, SUGANO H AND KITAGAWA T. (1992). Allelotype of non-small cell lung carcinoma – comparison between loss of heterozygosity in squamous cell carcinoma and adenocarcinoma. *Cancer Res.*, **52**, 2478–2481.

TSUTSUMI M, YOKOTA J, KAKIZOE T, KOISO K, SUGIMURA T AND TERADA M. (1989). Loss of heterozygosity on chromosomes 1p and 11p in sporadic pheochromocytoma. *J. Natl Cancer Inst.*, **81**, 367–370.

WEISS J, RUBINFELD B, POLAKIS PG, MCCORMICK F, CAVENEE WK AND ARDEN KC. (1994). The RAP1GA1 locus for human Rap1-GTPase activating protein 1 maps to chromosome 1p36.1–>p35. *Cytogenet. Cell Genet.*, **66**, 18–21.

WEITH A, MARTINSSON T, CZIEPLUCH C, BRUDERLEIN S, AMLER LC, BERTHOLD F AND SCHWAB M. (1989). Neuroblastoma consensus deletion maps to 1p36.1–2. *Genes Chrom. Cancer*, **1**, 159–166.

WESTON A, CAPORASO NE, PERRIN LS, SUGIMURA H, TAMAI S, KRONTIRIS TG, TRUMP BF, HOOVER RN AND HARRIS CC. (1992). Relationship of H-*ras*-1, L-*myc*, and p53 polymorphisms with lung cancer risk and prognosis. *Env. Health Persp.*, **98**, 61–67.

WESTON A, LING-CAWLEY HM, CAPORASO NE, BOWMAN ED, HOOVER RN, TRUMP BF AND HARRIS CC. (1994). Determination of the allelic frequencies of an L-*myc* and a p53 polymorphism in human lung cancer. *Carcinogenesis*, **15**, 583–587.

YOKOTA J, WADA M, YOSHIDA T, NOGUCHI M, TERASAKI T, SHIMOSATO Y, SUGIMURA T AND TERADA M. (1988). Heterogeneity of lung cancer cells with respect to the amplification and rearrangement of *myc* family oncogenes. *Oncogene*, **2**, 607–611.

YOUNG J, BUTTENSHAW R, BUTTERWORTH L, WARD M, SEARLE J, LEGGETT B AND CHENEVIX-TRENCH G. (1994). Association of the SS genotype of the L-*myc* gene and loss of 18q sequences with a worse clinical prognosis in colorectal cancers. *Oncogene*, **9**, 1053–1056.